# Bovine Serum Albumin-Glutaraldehyde Sealed Fish-Mouth Closure of the Pancreatic Remnant during Distal Pancreatectomy

**DOI:** 10.1155/2017/9747421

**Published:** 2017-01-17

**Authors:** Fritz Klein, Igor Maximilian Sauer, Johann Pratschke, Marcus Bahra

**Affiliations:** Department of Surgery, Charité-Universitätsmedizin Berlin, Augustenburger Platz 1, 13353 Berlin, Germany

## Abstract

*Introduction*. Postoperative pancreatic fistula formation remains the major complication after distal pancreatectomy. At our institution, we have recently developed a novel bovine serum albumin-glutaraldehyde sealed hand sutured fish-mouth closure technique of the pancreatic remnant during distal pancreatectomy. The aim of this study was to analyze the impact of this approach with regard to technical feasibility and overall postoperative outcome.* Patients and Methods*. 32 patients who underwent a bovine serum albumin-glutaraldehyde sealed hand sutured fish-mouth closure of the pancreatic remnant during distal pancreatectomy between 2012 and 2014 at our institution were analyzed for clinically relevant postoperative pancreatic fistula formation (Grades B and C according to ISGPF definition) and overall postoperative morbidity.* Results*. Three out of 32 patients (9.4%) developed Grade B pancreatic fistula, which could be treated conservatively. No Grade C pancreatic fistulas were observed. Postpancreatectomy hemorrhage occurred in 1 patient (3.1%). Overall postoperative complications > Clavien II were observed in 5 patients (15.6%). There was no postoperative mortality.* Conclusion*. The performance of a bovine serum albumin-glutaraldehyde sealed hand sutured fish-mouth closure of the pancreatic remnant was shown to be technically feasible and may lead to a significant decrease of postoperative pancreatic fistula formation after distal pancreatectomy.

## 1. Introduction

Distal Pancreatectomy (DP) is performed as the standard procedure in patients with malignant, cystic, or neuroendocrine tumors and/or chronic pancreatitis in the body and tail of the pancreas [1]. Continuous progress in peri- and postoperative management as well as in surgical expertise has led to a decline in operation associated mortality to 0–7% even in patients with an advanced stage of disease in which DP was combined with additional vascular or visceral resections [2–4]. Postoperative morbidity after DP however remains high and is reported to range from 35% to 60% [5, 6]. Clinically relevant postoperative pancreatic fistula formation (POPF) Grades B and C which occur in up to 21% of all patients especially have a major impact on postoperative outcome and may lead to additional complications such as delayed gastric emptying, intra-abdominal abscess formation, sepsis, or hemorrhage from major visceral vessels and may therefore contribute not just to a prolonged hospital stay but eventually to a fatal postoperative outcome [5, 7–10]. The surgical technique as well as the status of the pancreatic remnant (soft versus hard parenchyma), patient age and body-mass index, patient care at a high-volume center, chronic pancreatitis, and the extent of lymphadenectomy, and visceral resection have been identified as predicting factors for POPF [7, 11, 12]. Numerous surgical techniques and variants for pancreatic remnant closure have been described for possible POPF reduction including hand-sewn suturing, various stapler methods, or a combination of both. Furthermore, pancreatoenteric anastomosis or the application of fibrin sealants or meshes, falciform ligament or gastric serosa patches, or the use of saline-coupled bipolar electrocautery and ultrasonic dissection had been evaluated [13–20]. However, no gold-standard technique with regard to POPF reduction has been established yet. Inspired by the well-established use of bovine serum albumin-glutaraldehyde (BioGlue®) sealing in cardiovascular surgery we have recently developed a novel technique of a bovine serum albumin-glutaraldehyde sealed fish-mouth closure of the pancreatic remnant during distal pancreatectomy in attempt to further reduce POPF. BioGlue (25% bovine serum albumin and 10% glutaraldehyde; Cryolife Inc., Kennesaw, GA, USA) was approved by the FDA in 1998 for surgical application and is utilized in cardiovascular and pulmonary surgery, that is, for application during aortic root reconstruction and valve placement as well as for control of alveolar air leaks [21, 22]. Clinical and histopathological studies have demonstrated that this semisynthetic surgical adhesive upon application polymerises with native tissues including pancreatic tissue and thereby creates a flexible mechanical seal which strengthens and holds tissues together with an additive haemostatic property [23, 24]. The aim of this study was to analyze the technical feasibility of this novel surgical approach as well as to investigate a possible benefit with regard to POPF and overall postoperative outcome.

## 2. Patients and Methods

We retrospectively analyzed 33 consecutive patients who underwent a bovine serum albumin-glutaraldehyde (BioGlue) sealed fish-mouth closure of the pancreatic remnant during distal pancreatectomy for primary malignant or cystic tumors of the pancreas as well as for chronic pancreatitis at the Department of General, Visceral and Transplantation Surgery, Charité-Universitätsmedizin Berlin, Campus Virchow, between January 1, 2012, and January 1, 2015. All operations were performed by five experienced visceral surgeons who were all educated about the technical steps of this novel procedure as part of the inclusion criteria of this study. This study was performed in accordance with the Declaration of Helsinki and its amendments and approved by the institutional ethic committee.

### 2.1. Surgical Technique

**Figure 1:**
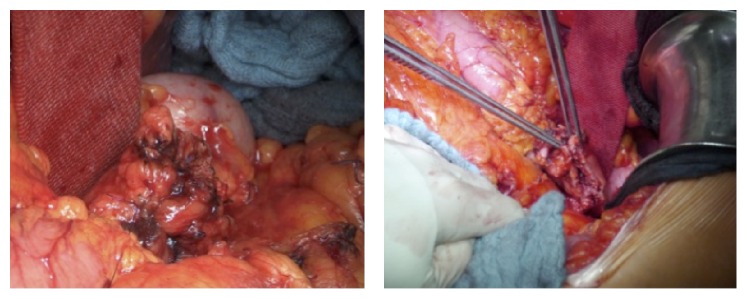
Fish-mouth shaped cutting surface of the pancreatic remnant.

**Figure 2:**
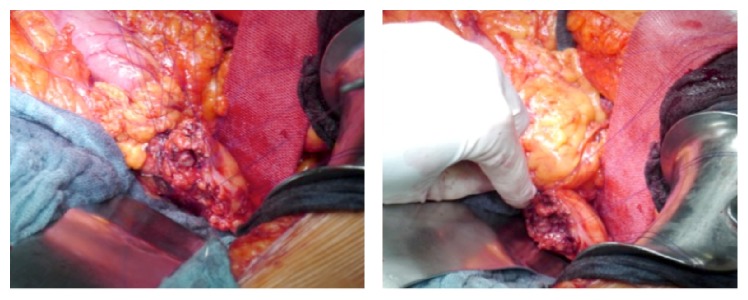
Single U-shaped 4-0 Prolene sutures placed along the cutting surface.

**Figure 3:**
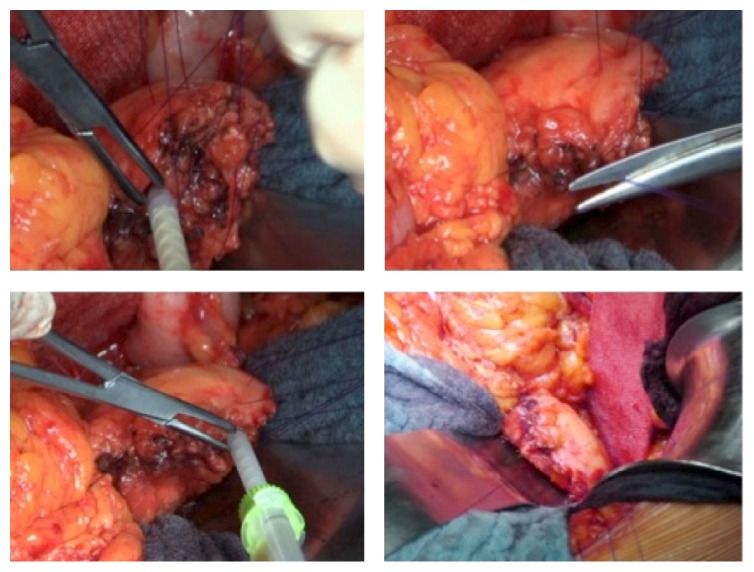
Bovine serum albumin-glutaraldehyde (BioGlue) administration into the fish-mouth cavity of the pancreatic remnant before the tying of each suture.

**Figure 4:**
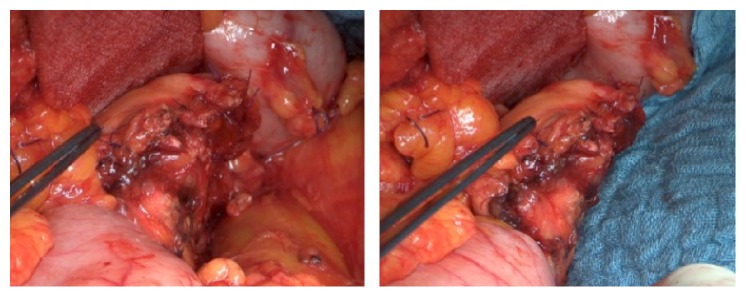
Pancreatic remnant after bovine serum albumin-glutaraldehyde sealed fish-mouth closure.

DP was performed as open surgery in each patient. The extent of the pancreatic resection, as well as the need for additional lymphadenectomy and splenectomy, was determined based on the underlying disease and/or cancer stages. The pancreatic resection was performed in all cases using electrocautery in an incision line, which creates a fish-mouth shaped cutting surface of the pancreatic remnant (Figure 1). After achievement of local hemostasis, subsequent closure of the main pancreatic duct (MDP) was performed by a stitch ligation using 4-0 polypropylene sutures (Prolene, Johnson & Johnson Medical GmbH, Norderstedt, Germany). As a next step single U-shaped 4-0 polypropylene sutures were placed along the cutting surface (Figure 2). The bovine serum albumin-glutaraldehyde (BioGlue, Cryolife Inc., Kennesaw, GA, USA) was then administered into the fish-mouth cavity of the pancreatic remnant in a step-by-step approach before the tying of each suture (Figure 3). No additional covering of the pancreatic remnant was performed (Figure 4). An intra-abdominal drain was placed at the pancreatic stump and if additional splenectomy was performed another intra-abdominal drain was positioned against the left subdiaphragmatic region.

### 2.2. Standard Postoperative Care

The levels of amylase and/or lipase in the blood and in the intra-abdominal drains were routinely measured on the 2nd and 5th postoperative day or immediately in the presence of laboratory or clinical signs of infection. Oral food intake was usually begun on the second postoperative day depending on the patient condition and bowel function. The drains were usually removed within 6 postoperative days if the output was clear and if there were no signs or symptoms of infection. We did not perform a standardized Somatostatin analogue (e.g., octreotide) prophylaxis for POPF prevention and pancreatic enzyme were only supplemented in the event of clinical signs of exocrine pancreatic insufficiency.

We also did not use a standardized protocol for POPF treatment. The drains were left in situ or replaced interventionally and continuous rinsing of the drain was initiated until the drain secretion was clear and the amylase/lipase had declined back to normal values. A cessation of oral food intake and/or eventually octreotide administration during POPF treatment was decided on individual basis in each patient.

The patients who had undergone an additional splenectomy received haemophilus, pneumococcal, and meningococcal vaccination according to current guidelines [25].

All patients were observed in our outpatient department for a postoperative period of at least 30 days.

### 2.3. Data Collection and Study Endpoints

Data collection in all patients included relevant information on their medical history, the pathological examination with regard to the underlying disease and resection margin status and the overall postoperative clinical outcome with documentation of any significant procedure related morbidity, the need for reintervention or reoperation, and the length of hospital stay. Postoperative morbidity was classified according to the Clavien-Dindo classification [26]. POPF and postpancreatectomy hemorrhage (PPH) were defined based on the ISGPF definitions [27, 28].

Besides a general analysis of the technical feasibility of this novel approach the primary outcome of our study was to investigate the rate of clinically relevant POPF equivalent to POPF Grades B and C. All other complications within 30 days of the operation were also recorded.

### 2.4. Statistical Analysis

Statistical analysis was performed using PASW statistics 19 (SPSS Software, IBM Company, Chicago, IL, USA). Continuous variables were reported using mean or median values where appropriate with range, whereas categorical variables were described using frequencies and percent.

## 3. Results

### 3.1. Patient Baseline and Preoperative Data

Between January 1, 2012, and January 1, 2015, 32 consecutive patients underwent a bovine serum albumin-glutaraldehyde sealed fish-mouth closure of the pancreatic remnant during distal pancreatectomy.

There were 15 males (47%) and 17 females (53%) with a median age of 62 years (32–77). The indication for distal pancreatectomy was pancreatic adenocarcinoma in 11 patients (34%), intraductal papillary mucinous neoplasm (IPMN) of the pancreas in 7 patients (22%), chronic pancreatitis in 3 patients (9%), neuroendocrine tumors (NET) of the pancreas in 2 patients (6%), mucinous cystic neoplasms (MCN) of the pancreas in 3 patients (9%), cyst adenoma in 4 patients (13%), cyst adenocarcinoma in 1 patient (3%), and leiomyosarcoma in 1 patient (3%). Two of the 32 patients (6%) underwent endoscopic retrograde cholangiopancreatography (ERCP) with endoscopic papillotomy (EPT) and endoscopic pancreas stent placement prior to the operation (Table 1).

### 3.2. Peri- and Postoperative Course

A splenectomy was performed in 27 of the 32 patients (84%). Seven patients (22%) underwent an additional multivisceral resection with partial or total gastrectomy in 4 patients (13%), colon resection in 5 patients (16%), and/or partial adrenalectomy in 3 patients (9%). The mean operation time was 199 minutes (116–282 minutes). No patient required intraoperative administration of packed red blood cells. The pancreas tissue texture was found to be soft in 13 patients (41%) and hard in 19 patients (59%) according to the intraoperative assessment of the operating surgeon. Major postoperative morbidity > Clavien II occurred in 5 of the 32 patients (16%). Three patients (9%) developed a clinically relevant POPF Grade B. Each of the three patients could be treated conservatively with a however prolonged hospital stay of 40, 71, and 93 days. No Grade C pancreatic fistulas were observed. Postpancreatectomy hemorrhage Grade A occurred in one patient (3%) as an intraluminal bleeding, which did not require therapeutic consequences. Two patients underwent reoperations, due to an insufficiency of a colon anastomosis in one patient and an abdominal fascial dehiscence in another patient. There was no postoperative mortality. The median length of hospital stay was 12 days (7–93) (Table 2).

## 4. Discussion

Postoperative pancreatic fistula formation remains the most relevant complication after distal pancreatectomy with a major impact on postoperative quality of life as well as on health care costs. In patients with an underlying malignant disease POPF may in addition lead to a delayed onset of further essential adjuvant treatment which has been identified as an independent risk factor for early peritoneal recurrence and decreased overall survival [29].

Numerous surgical techniques have therefore been described in an attempt to possibly decrease the incidence and impact of POPF. However, even two recent prospective randomized trials failed to identify an ideal technique for the procedure with the pancreatic remnant. The DISPACT trial compared a stapler versus hand-sewn closure of the pancreatic remnant and the incidence of clinically relevant POPF Grades B and C was reported with 20% and 21% in each group [5]. Analogously Carter et al. analyzed the effect of the use of an autologous falciform ligament patch with fibrin glue for both stapler and hand-sewn closure of the pancreatic remnant and reported a rate of clinically relevant POPF Grades B and C in 18% of their patients which did not differ significantly from the control group [30]. The question of an optimal surgical technique for distal pancreatectomy therefore remains to be debated controversially. At our institution we have recently developed a novel technique of a bovine serum albumin-glutaraldehyde (BioGlue) sealed fish-mouth closure of the pancreatic remnant during distal pancreatectomy, which was analyzed in this study. We could demonstrate that our technique was feasible with a distinct positive impact on clinically relevant POPF. Only three of our 32 patients (9%) developed a POPF Grade B, which could be treated conservatively, and no POPF Grade C was observed. With a clinically relevant POPF rate below 10% our results are thus to be seen promising especially in comparison to the results of the present prospective as well as retrospective studies [5, 13–15, 17–20, 30]. The concept of bovine serum albumin-glutaraldehyde application in pancreatic surgery is not new. Fisher et al. have previously investigated the use of BioGlue in an attempt to possibly reduce POPF and demonstrated general safety but no relevant clinical benefit [31]. Most patients in this study however underwent pancreaticoduodenectomy (PD) instead of DP and the bovine serum albumin-glutaraldehyde was only applied on the completed anastomosis. We modified this technique by creating a fish-mouth shaped pancreatic remnant with a ligated main pancreatic duct (MPD). The bovine serum albumin-glutaraldehyde (BioGlue) was then applied into the fish-mouth cavity before the tying of previously placed single U-shaped sutures. Analogously to Ohwada et al. we believe that our technique enables the bovine serum albumin-glutaraldehyde adhesive to stay within the anastomosis region as opposed to the external application described by Fisher et al. in which the BioGlue may rather likely dissolve [32].

The creation of a fish-mouth shaped cutting margin of the pancreatic remnant may of course be challenging especially in a rather thin gland and may also be associated with an increased bleeding tendency [33]. Also the identification and routinely performed ligation of the main pancreatic duct which is considered an individual factor for POPF reduction may not always be possible [34]. We did however not experience any intraoperative problems of this kind.

The use of bovine serum albumin-glutaraldehyde (BioGlue) as a sealant is of course rather expensive especially in comparison to widely used fibrin glue. However, several studies showed that the use of fibrin for sealing the cutting surface after PD or DP as well as for occlusion of the main pancreatic duct was not associated with a reduction of POPF [18, 35]. In addition to the limited clinical evidence fibrin may be considered a poor adhesive for pancreatic surgery because it takes a long time to set up and may therefore be swiped or washed away easily. Bovine serum albumin-glutaraldehyde in contrast reaches maximal strength within 2 minutes after application with an additional benefit of local hemostatic properties [31]. We believe that a quick transformation of the liquid glue to a flexible hydrogel is especially important in this setting to prevent a loss of attachment to the applied surface.

Complications of bovine serum albumin-glutaraldehyde (BioGlue) application reported in cardiovascular and pulmonary surgery include nerve damage, local or embolic vascular obstruction, and foreign body reactions [36, 37]. Lämsä et al. have previously analyzed the histological effects of tissue adhesives on the pancreas and reported acinar cell vacuolization and necrosis together with moderate edema and leukocyte infiltration in each adhesive tested with however no relevant differences between fibrin and bovine serum albumin-glutaraldehyde (BioGlue) [24]. In our study we did not observe any of such complications reaching a clinical manifestation especially no clinical or laboratory signs of a postoperative pancreatitis.

It should of course be noted that any kind of additional physical barrier may lead to a delayed onset or at least clinical presentation of POPF [30]. As part of our internal treatment standard patients were however only discharged if all parameters indicating infection were low and the patient was feeling well which is reflected by a prolonged median hospital stay of 12 days in our study. In addition patients were observed in our outpatient department for a postoperative period of at least 30 days. A potentially undetected delayed onset of POPF may thus be excluded in our patient population.

A reduction in the incidence of POPF may of course not just be achieved by restricting on surgical technical factors alone. Several studies have reported an improved postoperative outcome if EPT or pancreatic stent placement were performed prior to DP [38]. This potential benefit is however outweighed by an increased risk for directly intervention related morbidity which is reported to occur in up to 57% of all patients [39]. In our study ERCP with EPT and/or stent placement was thus only performed in 2 of our 32 patients (6%) and only considered in patients with suspected benign diseases in an attempt to avoid any additional risk for a potential delayed onset of treatment in patients with an underlying malignant disease. The statistical power of our study is of course limited by the small sample size of patients. Also the retrospective study design may have led to an unintended selection bias. However, as a conclusion of our study we could demonstrate that our technique of a bovine serum albumin-glutaraldehyde sealed fish-mouth closure of the pancreatic remnant was technically feasible and safe with a high potential to decrease the incidence as well as the impact of clinically relevant POPF after DP.

## Figures and Tables

**Table 1:** Demographics and preoperative characteristics.

	BioGlue sealed hand sutured fish-mouth closure technique during distal pancreatectomy
Number of patients	32
Median age (years; range)	62 (32–77)
Gender (male : female)	15 (47%) : 17 (53%)
Indication	
Pancreatic adenocarcinoma	11 (34%)
IPMN	7 (22%)
Chronic pancreatitis	3 (9%)
NET	2 (6%)
MCN	3 (9%)
Cyst adenoma	4 (13%)
Others	2 (6%)
Preoperative ERCP with EPT and pancreatic stent placement	2 (6%)

**Table 2:** Operative data and clinical outcome.

	BioGlue sealed hand sutured fish-mouth closure technique during distal pancreatectomy (*n* = 32)
Mean operation time (minutes; range)	199 (116–282)
Splenectomy	27 (84%)
Additional visceral resection	7 (22%)
Partial/total gastrectomy	4 (13%)
Colon resection	5 (16%)
Partial adrenalectomy	3 (9%)
Pancreas tissue texture	
Soft	13 (41%)
Hard	19 (59%)
Postoperative morbidity > Clavien II	5 (16%)
Clinically relevant POPF	3 (9%)
POPF Grade B	3 (9%)
POPF Grade C	0
PPH	1 (3%)
Reoperation	2 (6%)
Median hospital stay (days; range)	12 (7–73)
Mortality	0
